# Factors associated with utilization of skilled service delivery among women in rural Northern Ghana: a cross sectional study

**DOI:** 10.1186/s12884-017-1344-2

**Published:** 2017-05-31

**Authors:** William Gudu, Bright Addo

**Affiliations:** 1 Bongo District Hospital, Bolgatanga, Upper East Region Ghana; 2 Population Council, Ghana Office, 14B, Ridge Road, Roman Ridge, P.O. Box CT 4906, Accra, Ghana

**Keywords:** Maternal mortality ratio, Antenatal care, Skilled delivery services, Health facilities, Rural district

## Abstract

**Background:**

Ghana’s current Maternal Mortality Ratio (MMR) of 319 per 100,000 live births makes achievement of the Sustainable Development Goal of 70 maternal deaths per 100,000 live births or less by 2030 appear to be illusory. Skilled assistance during childbirth is a critical strategy to reducing maternal mortality, yet the proportion of deliveries taking place within health facilities where such assistance is provided is very low in Ghana, with huge disparity between urban and rural women. To address the gap in skilled attendance in rural Upper East Region, the Ghana Health Service (GHS) in 2005 piloted a program that involved training of Community Health Officers (CHOs) as midwives. This study explored factors associated with skilled delivery services utilization in a predominantly rural district in Ghana.

**Methods:**

A cross-sectional study, data was collected from a sample of 400 women between the ages of 15 and 49 years who had given birth a year prior to the study. We used frequencies and percentages for descriptive analysis and chi-square (*χ* ^*2*^) test for relationship between independents factors and utilization of skilled delivery services.

**Results:**

Of the 400 women included in the analysis, 93.3% of them delivered in a health facility. Almost all of the mothers (97.3%) attended or received antenatal care at their last pregnancy with 75.0% of them having four or more ANC visits. The proportion of women who received ANC and utilized skilled delivery services was high (91.5%). Mother’s educational attainment, ANC attendance, frequency of ANC visits, satisfaction with ANC services and possession of valid NHIS card significantly associated with utilisation of skilled delivery services.

**Conclusion:**

For a predominantly rural district, the percentage of women who deliver within health facilities where skilled assistance is available is very encouraging and a significant stride towards reducing Ghana’s overall MMR. Having four or more ANC visits and improving on the quality of care provided has a great potential of improving uptake of skilled delivery services.

**Electronic supplementary material:**

The online version of this article (doi:10.1186/s12884-017-1344-2) contains supplementary material, which is available to authorized users.

## Background

Given the unacceptably high burden of mortality and disability in women of reproductive age, 189 Member states of the United Nations (UN) in 2000 pledged to work towards achieving a set of Millennium Development Goals. One of these goals was to achieve by the year 2015, a target (MDG 5A) of three-quarters reduction in Maternal Mortality Ratio (MMR), especially in sub-Saharan Africa. This target and that of achieving universal access to reproductive health (MDG 5B) together formed the overall goal of improving maternal health [1].

To achieve the MDG 5 targets, various policies and programmes have been put in place by various countries. In Ghana for example, the government in 2005 implemented the user fee exemption policy to make maternal services offered in all public health institutions non-payable and thereby increase accessibility and utilization of skilled birth assistance by women especially of lower socio-economic status. The policy was first piloted in four of the most disadvantaged regions (Northern, Upper East, Upper West and Central) in 2003 and then extended nationwide in 2005 with the goal of giving all women free delivery care including caesarean sections [2].

Again, with the aim of providing health care to all by removing cost as a barrier to receiving healthcare, the Government of Ghana (GoG) idealized and created the National Health Insurance Scheme (NHIS) in 2013, which became fully operationalized in 2005 [2]. Due to lack of funding, the Ministry of Health discontinued the fee exemption policy in 2005, which was the same year the government fully operationalized the NHIS. This situation led to the creation of a loop-hole in the healthcare system whereby women who were not enrolled on the NHIS would be required to pay for maternal care. Owing to this, the Ministry of Health came out with a new Maternal Health Care Program in 2008 which served to ensure that women, who were enrolled on the NHIS and were confirmed to be pregnant, were exempted from paying for their healthcare out of pocket. Services that were catered for by the program included: six (6) antenatal care visits, delivery care including care for complication, two (2) post-natal care visits within a 6 weeks period after childbirth and care of infants up to 3 months of age [2].

Fifteen years down the lane, maternal morbidity and mortality continue to be a problem globally, with most of these deaths concentrated at the time of delivery and a huge disparity in reported cases existing between developed and developing countries [1, 3]. Recent estimates indicate that annual maternal deaths decreased by 43.0% from approximately 532,000 in 1990 to an estimated 303,000 in 2015. According to the World Health Organisation (WHO) developing regions account for approximately 99.0% (302000) of the global maternal deaths, with countries from sub-Saharan Africa alone accounting for roughly 66% (201000), followed by Southern Asia (66000) [1].

In Ghana Maternal Mortality Ratio (MMR) has been decreasing over the last two decades. Current estimates from the WHO puts the country’s MMR at 319 maternal deaths per 100,000 live births [1]. To reduce maternal mortality and improve on perinatal outcomes for early borns, strategies such as seeking skilled assistance during childbirth, effective postnatal care within the first 24 h of delivery and readily available, accessible and appropriate care in cases of complication have been identified. Of these strategies, the most critical is the ‘health-centre intrapartum care strategy’ where qualified skilled health personnel manage labour, effectively manage complications should they occur and there is a supportive referral system for specialised care when needed [4].

Generally, experts agree that about 20.0% of stillbirths or deaths due to intrapartum-related complications can be reduced if births are attended to by skilled personnel [1]. Worldwide, it is estimated that about one in every four births (25.0%) occur without the assistance of a skilled birth attendant. In low and middle-income countries, this figure translated into more than 40 million in 2015. Ninety percent (90.0%) of these happened in South Asia and sub-Saharan Africa [1]. In Ghana, recent estimates by the 2014 GDHS indicated that only 73.0% of births occurred in health facilities, of which the public sector accounted for the largest proportion. This percentage was an increase since the 2008 GDHS (57.0%). This implies that some 27.0% of women did not utilize health facilities, but rather for reasons such as cost, distance to health facility and concerns about quality of care delivered at home [5]. The survey further showed that close to three-quarters of births (74.0%) in Ghana occured with the assistance of a skilled health professional. The 74.0% comprised of 14.0% deliveries assisted by a doctor; 57.0% deliveries assisted by a nurse or midwife; and 3.0% deliveries assisted by a community health officer/nurse. Sixteen percent (16.0%) of births were delivered by a traditional birth attendant, 7.0% were assisted by a relative or other person and 3.0% of deliveries were not assisted by anyone. The survey also indicated only 60.0% of rural women were delivered by a skilled provider as compared to 90.0% in urban areas [5].

Studies on utilization of skilled delivery services have demonstrated that the decision to use such a service is influenced by multiple factors. Interestingly, while certain factors have been found to be significant in determining the use of skilled delivery services in some studies, these same factors were found to be insignificant in others [3]. This difference in study results notwithstanding, individual factors such as maternal age, education, marital status, parity, household factors also including family size, household wealth and community and environmental factors including region, community health infrastructure, available health facilities and distance to health facilities have been identified to operate in diverse contexts to determine place of delivery [4].

Kitui et al. analysing the 2008/2009 Kenya DHS demonstrated that living in urban areas, being wealthy, more educated, using antenatal care services optimally and lower parity strongly predicted where women delivered and so did region, ethnicity and type of infrastructure used [4]. In Ghana, a recent study by Amoakoh-Coleman et al. using the 2008 GDHS to identify the demographic, maternal and community determinants of skilled attendance at birth by women who attend at least one ANC during their term of pregnancy found factors such as wealth status, residency, complications with previous pregnancy, health insurance coverage and religion to be significant predictors of skilled attendance at delivery [6]. Whiles there is now ample research on skilled delivery service utilization, especially in sub-Saharan Africa, most of these studies have been limited to the analysis of secondary data. In Ghana in particular, little community-based studies exist and only a handful have solely focused on rural areas. As methods to improve maternal health have proven to be reasonably simple and inexpensive [7], the fact that maternal mortality still remains a risk factor for women in Ghana, especially in rural areas where utilisation of skilled delivery services has been found to be very low [5, 8], necessitated the conduct of this study to identify the factors that significantly associate with skilled delivery services utilisation among rural women in Ghana.

## Methods

### Study area

The study was conducted in the Bongo District, one of the 13 districts of the Upper East Region of Ghana. The district is predominantly rural (93.9% rural and 6.1%), has population of 84,545 of which 47.4% (40,074) are males and 52.6% (44,471) [9]. The district constitutes about 8.1% of the region’s population with an estimated fertility rate of 101.2 which indicates that some 101 children are born to 1000 women aged between 15 and 49 years per year. The district has a Total Fertility Rate (TFR) of 3.7 births per woman [9]. The Bongo district has in total 132 communities scattered in small dispersed settlements. The district can boast of only one hospital and four health centres. The hospital serves as a referral centre for the district’s population and neighbouring country Burkina Faso. This creates enamours pressure on the hospital and few heath centres in the district. With the introduction and implementation of the Community Health Planning and Services (CHPS) concept in the district, the pressure on these health facilities is anticipated to be reduced. Currently, there are seven (7) functional CHPS compounds which cover about 20.2% of the districts population. The major challenges to health care delivery in the district include inadequate health infrastructure (such as residential accommodation), inadequate health personnel and inadequate logistics and equipment [9, 10].

### Study design

The study was a cross-sectional survey employing quantitative methods. Women of reproductive ages 15-to-49 years who had given birth a year prior to the study responded to structured questionnaires.

### Population and sample size

The study population consisted of women of child bearing ages 15-to-49 years who had delivered within a period of 12 months (1 year) prior to the survey and resided in the district at the time of the study.

#### Sample size estimation

The Proportion of women who delivered at home in the Upper East Region by the GDHS in 2008 was estimated at 52.6% [8]. At 95% confidence interval and margin of error (d) of 5%, the sample size was determined using the formula:

$$ N=\frac{\mathrm{z}2\times \mathrm{p}\left(1-\mathrm{p}\right)}{\mathrm{d}2} $$

Where:

N = Target Population.

z = is standardized normal distribution curve value for the 95% confidence interval (1.96).

p = estimated prevalence of women delivering at home in study area (0.51).

d = margin of error at 5%.

n  =  (1.962)  ×  0.53(1 − 0.53)  ÷  0.052.

n  =  (3.8416  ×  0.53  ×  0.47)  ÷  0.052

n  =  0.960  ÷  0.0025

n = 384

10% non-response rate added to give a total of 422 participants.

### Sampling procedure

A stratified sampling method was employed to first select the sub-districts. Based on the sub-district categorization by the 2010 Population and Housing Census, 6 sub-districts namely; Bongo Central, Soe, Beo, Namoo, Zorko and Valley View were selected. Next was the selection of communities under these 6 sub-districts. The Bongo district according to the 2010 PHC District Analytical Report is made of 132 communities. The non-existence of information on the number of communities in each of these sub-districts led to selection of nineteen (19) (out of 20) of the largest communities identified by the analytical report. For representativeness, twenty-two (22) eligible respondents in each community were estimated to be interviewed based on the sample size of 422 computed. Final sampling was the selection of households. The first household on the right as one entered each community was selected as the starting point, after which every fifth household was selected. Women who had children up to 1 year in a household were interviewed (administered with the questionnaire). In households with more than one eligible respondent, the number of respondents who were willing to participate engaged in a ballot and one of them selected. Where there was no eligible respondent (recently delivered mother), the research assistants moved on to the next household until an eligible respondent was found. A total of 418 mothers participated in the study.

### Variables and measurement

The dependent variable was place of last delivery which was categorized into “health facility delivery” or “non-health facility delivery”. Health facility delivery was operationalized as delivery occurring in a public or private hospital/clinic/maternity home, a health centre and a CHPS compound. Non-health facility delivery was also operationalized as a delivery occurring outside of a health facility which included being delivered by a Traditional Birth Attendant (TBA), woman delivering at home or on her way to a health facility. The independent variables measured by the study were categorized as background characteristics of respondents, antenatal care history of women and their socio-economic characteristics. Background characteristics of respondents measured included age, parity, marital status, ethnicity, religion, educational attainment of mother and partner’s educational attainment. Antenatal care history consisted of ANC receipt, place where ANC was received, number of months pregnant on receipt of first ANC, frequency of ANC visits and satisfaction with ANC services received. Socio-economic characteristics included person who influenced respondent’s decision on place of delivery, possession of a valid NHIS card, means of transportation to place of delivery, and wealth status. Assets used to measure the wealth quintile included 6 items; ownership of a car, refrigerator and/or freezer, radio, television, stove and telephone/cell phone.

### Questionnaire validation

The first step in the questionnaire validation process was to establish face validity. Two experts, a University Professor and a practicing Gynaecologist who understood the topic read through the draft questionnaire and evaluated it to ascertain if the questions asked effectively captured the topic under investigation. Their suggestions and recommendations made were then incorporated. After the face validation process, the questionnaire was pre-tested in one of the communities in the district. Thirty women with similar characteristics as the study sample were administered with the questionnaire. Appropriate revision and rephrasing of questions were made based on the outcome of results from the pre-test for final use.

### Data collection and analysis

The data was collected using structured questionnaires between August and September, 2013. The questions were asked in the local language Frafra, however responses were recorded in English for easy analysis. To ensure quality and validity of the data, research assistants were recruited and trained. During the data collection period, research assistants were monitored through regular unannounced visits to the study areas. This was to ensure that the relevant data were collected. Each day, data were checked for completeness to ensure that all information were properly collected and recorded. Errors and omissions detected were discussed with the respective research assistants and were made to go back and make the necessary corrections. Out of the 418 questionnaires received from research assistants, eighteen (18) were rejected leaving 400 for data entry and analysis. The data once received was coded, entered, cleaned and analysed using the Statistical Package for Social Sciences (version 20) software programme. Descriptive analysis was conducted on respondents’ background characteristics and reported in frequencies and percentages. Relational tests between independent and dependent variables were performed using the chi-square test of independence (χ^2^).

### Ethical considerations

The study obtained ethical approval from the Ethical Review Committee of the Ghana Health Service through the University of Ghana School of Public Health. Approval was also obtained from the Upper East Regional Health Directorate (RHD) and Bongo District Health Management Team (DHMT) before data was collected. Respondents were informed of the purpose of the study after which they signed a consent form indicating their volition to participate in the study as well as assurance of their confidentiality and privacy. It was stressed that participation was voluntary and any participant who wished to withdraw from the study was allowed to do so. The study was not supported by any external source of funding nor compensation or monetary benefit given to participants who took part in the study. The study had no conflict of interest.

## Results

### Background characteristics of study respondents

Data from 400 respondents were analysed. Up to 373 (93.3%) women delivered in health facilities. The mean age of the women was 27 (SD=6.3) years, with most of them between the ages of 24 and 29 years. Majority of the women had between 1 and 2 children. About 80% (79.8%) of the women were currently married during the survey. Majority of the women were also Frafras (83.0%). The dominant religions were Christianity (63.0%) and Traditional African faith (27.3%). Forty-one percent (41.0%) of women and 38.8% of their partners had no formal education (Table 1).

**Table 1:** Descriptive data on background characteristics of respondents

Characteristics	Frequency (%) (*N* = 400)	Non- health facility delivery (*N* = 27)	Health facility delivery (*N* = 373)
		n (%)	n (%)
Age of mother			
16–25	178 (44.5)	8 (2.0)	170 (42.5)
20–29	173 (43.3)	15 (3.8)	158 (39.5)
30–39	49 (12.3)	4 (1.0)	45 (11.3)
Parity			
1–2	204 (51.0)	10 (2.5)	194 (48.5)
3–4	148 (37.0)	13 (3.3)	135 (33.7)
5+	48 (12.0)	4 (1.0)	44 (11.0)
Marital status			
Married	319 (79.8)	20 (5.0)	299 (74.8)
Cohabiting	2 (0.5)	0 (0.0)	2 (0.5)
Divorced/separated/widowed	30 (7.5)	1 (0.3)	29 (7.2)
Never married	49 (12.3)	6 (1.5)	43 (10.8)
Ethnicity			
Frafra	332 (83.0)	23 (5.8)	309 (77.2)
Kusa	2 (0.5)	0 (0.0)	2 (0.5)
Gruni	66 (16.5)	4 (1.0)	62 (15.5)
Religion			
Christian	252 (63.0)	16 (4.0)	236 (59.0)
Islam	39 (9.8)	0 (0.0)	39 (9.8)
Traditional	109 (27.3)	11 (2.8)	98 (24.5)
Educational attainment of mother			
None	164 (41.0)	17 (4.3)	147 (36.7)
Primary	107 (26.8)	7 (1.8)	100 (25.0)
Middle/JSS^a^/JHS^b^	96 (24.0)	2 (0.5)	94 (23.5)
Secondary/SSS^c^/SHS^d^/Tech^e^/Voc^f^	28 (7.0)	0 (0.0)	28 (7.0)
Tertiary	5 (1.3)	1 (0.3)	4 (1.0)
Educational attainment of partner			
None	155 (38.8)	16 (4.0)	139 (34.8)
Primary	46 (11.5)	2 (0.5)	44 (11.0)
Middle/JSS^a^/JHS^b^	65 (16.3)	0 (0.0)	65 (16.3)
Secondary/SSS^c^/SHS^d^/Tech^e^/Voc^f^	28 (7.0)	1 (0.3)	27 (6.7)
Tertiary	25 (6.3)	1 (0.3)	24 (6.0)
Not Applicable	81 (20.3)	7 (1.8)	74 (18.5)

^a^ *JSS* Junior Secondary School, ^b^ *JHS* Junior High School, ^c^ *SSS* Senior Secondary School, ^d^ *SHS* Senior High School, ^e^ *Tech* Technical School, ^f^ *Voc* Vocational School

### Antenatal care history

Almost all of the mothers (97.3%) attended or received antenatal care at their last pregnancy. Majority of the mothers who patronized ANC services did so at a health center (71.8%). The majority of mothers (51.8%) had their first ANC in the first and second month (first trimester) of their pregnancy. Three quarters (75.0%) of the women had four or more ANC visits. Almost all (96.0%) women who received ANC were satisfied with the care received. The proportion of women who received ANC and utilized skilled delivery services was high (91.5%). Majority of the women who had four or more ANC visits and those who were satisfied with the care provided delivered at a health facility (69.7% and 90.7% respectively) (Table 2).

**Table 2:** Antenatal care (ANC) history of respondents

Variable	Frequency (%) (*N* = 400)	Non- health facility delivery (*N* = 27)	Health facility delivery (*N* = 373)
		n (%)	n (%)
Received ANC at last birth			
Yes	389 (97.2)	23 (5.8)	366 (91.5)
No	11 (2.8)	4 (1.0)	7 (1.8)
Place of ANC			
Hospital	82 (20.5)	4 (1.0)	78 (19.5)
Health center	287 (71.8)	16 (4.0)	271 (67.8)
Outreach point	20 (5.0)	3 (0.8)	17 (4.2)
Not applicable	11 (2.8)	4 (1.0)	7 (1.8)
Months pregnant at first ANC			
< 3	207 (51.8)	12 (3.0)	195 (48.8)
3–6	124 (31.0)	9 (2.3)	115 (28.7)
> 6	19 (4.8)	1 (0.3)	18 (4.5)
Don’t know	39 (9.8)	1 (0.3)	38 (9.5)
Not applicable	11 (2.8)	4 (1.0)	7 (1.8)
Number of ANC visits			
One	6 (1.5)	0 (0.0)	6 (1.5)
Two	12 (3.0)	0 (0.0)	12 (3.0)
Three	71 (17.8)	2 (0.5)	69 (17.3)
Four or more	300 (75.0)	21 (5.3)	279 (69.7)
Not applicable	11 (2.8)	4 (1.0)	7 (1.8)
Satisfaction with ANC provided			
Yes	384 (96.0)	21 (5.3)	363 (90.7)
No	5 (1.2)	2 (0.5)	3 (0.7)
Not applicable	11 (2.8)	4 (1.0)	7 (1.8)

### Socio-economic characteristics

Close to half of the women (47.0%) who delivered within health facilities indicated that it was their own preference/choice. Most of the women also indicated that it was a joint decision they made with their partner. Majority of women who delivered within health facilities walked to the place. Almost all the women (96.0%) possessed valid health insurance, with majority of these women (86.7%) also delivering within health facilities. Majority of the study respondents were less wealthy, that is, belonged to the lower wealth quintile (Table 3).

**Table 3:** Socio-economic characteristics of respondents

Variable	Frequency (%) (*N* = 400)	Non- health facility delivery (*N* = 27)	Health facility delivery (*N* = 373)
		n (%)	n (%)
Influence of women’s decision on delivery at health facility			
Own preference/choice	188 (47.0)	-	188 (47.0)
Collective decision with partner	138 (34.5)	-	138 (34.5)
Collective decision by family	46 (11.5)	-	46 (11.5)
Means of transportation to delivery venue			
Walk	325 (81.3)	23 (5.8)	302 (75.5)
Taxi or bus	10 (2.5)	0 (0.0)	10 (2.5)
Own car	8 (2.0)	0 (0.0)	8 (2.0)
Motor cycle	34 (8.5)	1 (0.3)	33 (8.2)
Bicycle	23 (5.8)	3 (0.8)	20 (5.0)
NHIS valid card holder			
Yes	384 (96.0)	23 (5.8)	361 (90.2)
No	16 (4.0)	4 (1.0)	12 (3.0)
Wealth quintiles			
Less wealthy	374 (93.5)	27 (6.8)	347 (86.7)
Wealthy	20 (5.0)	0 (0.0)	20 (5.0)
Wealthier	6 (1.6)	0 (0.0)	6 (1.6)

### Factors associated with utilization of skilled delivery services

Results of the bivariate analysis conducted using the chi-square test of independence revealed that mother’s educational attainment, ANC attendance, frequency of ANC visits, satisfaction with ANC services and possession of valid NHIS significantly related to women’s use of skilled delivery services (Table 4).

**Table 4:** Bivariate analysis of factors that associate with use of skilled delivery services

Characteristics	Non- health facility delivery (*N* = 27)	Health facility delivery (*N* = 373)	*p*-value for χ^2^ test
	n (%)	n (%)	
Age of mother			
16–25	8 (4.5)	170 (95.5)	.271
26–35	15 (8.7)	158 (91.3)	
36–45	4 (8.2)	45 (91.8)	
Parity			
1–2	10 (4.9)	194 (95.1)	.321
3–4	13 (8.8)	135 (91.2)	
5+	4 (8.3)	44 (91.7)	
Marital status			
Married	20 (6.3)	299 (93.7)	.366
Cohabiting	0 (0.0)	2 (100.0)	
Divorced/separated/widowed	1 (3.3)	29 (96.7)	
Never married	6 (12.2)	43 (87.8)	
Educational attainment of mother			
None	17 (10.4)	147 (89.6)	.038*
Primary	7 (6.5)	100 (93.5)	
Middle/JSS/JHS	2 (2.1)	94 (97.9)	
Secondary/SSS/SHS/Tech/Voc	0 (0.0)	28 (100.0)	
Tertiary	1 (20.0)	4 (80.0)	
ANC attendance^a^			
Yes	23 (5.9)	366 (94.1)	.000***
No	4 (36.4)	7 (63.6)	
Number of ANC visits^a^			
One	0 (0.0)	6 (100.0)	001**
Two	0 (0.0)	12 (100.0)	
Three	2 (2.8)	69 (97.2)	
Four or more	21 (7.0)	279 (93.0)	
Satisfaction with ANC provided^a^			
Yes	21 (5.5)	363 (94.5)	.000***
No	2 (40.0)	3 (60.0)	
NHIS valid card holder			
Yes	23 (6.0)	361 (94.0)	.003**
No	4 (25.0)	12 (75.0)	
Wealth quintile			
Less wealthy	27 (7.2)	347 (92.8)	.366
Wealthy	0 (0.0)	20 (100.0)	
Wealthier	0 (0.0)	6 (100.0)	

^a^Response category excludes Not applicable response

*p < .05, **p < 0.01, ***p < .001

## Discussion

Pregnancy and childbirth are inextricably a part of the life of most women. While this is ordinarily a period of pride and joy, it is associated with great pain, disability and even death for too many women particularly in developing countries [11]. Most of these deaths and disabilities can be prevented through access to and utilization of maternal health care services [11–13]. Broadly this study was dedicated to exploring the factors that associate with delivery in health facilities in a predominantly rural district in Northern Ghana. In addition, the study investigated the antenatal care history of respondents.

Findings of the study on ANC coverage suggest the availability and high uptake of antenatal care services among pregnant women in the Bongo district. Generally higher uptake of ANC is seen in most countries compared with delivery care. Two key reasons have been cited for this and they are: (1) the greater availability of antenatal services at the various levels of health facilities including mobile services in some countries, and (2) the relatively low cost of providing ANC services compared with delivery care services [14]. Additionally, unlike ANC, the precise timing of a delivery is unpredictable and this makes it more difficult for expectant women without access or with restricted access to transportation to reach delivery care [14]. The purpose of ANC is to prevent or identify and treat conditions that may threaten the health of a mother or the foetus and to help expectant mothers approach pregnancy and birth as positive experiences. The WHO recommends for normal pregnancies, just four ANC visits [6, 12, 15]. Majority of the women who participated in the study had four or more antenatal visits which satisfy the WHO requirement of at least just four ANC visits. Antenatal care visits accrue beneficially to the health and survival of the mother and infant [13, 14].

The trend presented in national as well as regional figures showing consistently high uptake of antenatal care services and on the converse low uptake of skilled delivery services did not hold true for findings from the Bongo district. There was only a 5.8% difference in the percentage of women who received ANC (97.3%) and those who delivered at a health facility (91.5%). The high uptake of skilled delivery service in the district could be attributed to the piloting of the Ghana Health Service program which involved training Community Health Officers (CHOs) as midwives in rural Upper East Region, and the high quality of care provided in the district. The GHS in 2005 piloted a program that involved training of CHOs as midwives to address the gap in skilled attendance in rural Upper East Region. The Bongo district was a beneficiary of this program which helped to boost the number of health personnel offering skilled delivery services in the district [16].

An evaluative study conducted by the Alliance for Reproductive Health Rights in 2014 in four districts (i.e. Agona East and Komenda-Edina-Eguafo-Abirem (KEEA) districts in the Central Region, and Bongo and Builsa Districts in the Upper East Region) of Ghana towards progress made with achieving the MDGs 4 and 5, indicated that, in the Bongo and Builsa districts, where high scores were recorded for quality of care, respondents described health care workers as caring and they gave prompt attention to mothers. Such good attitude of health workers contributed to high satisfaction with the services provided. Further, the report indicated that in Bongo, the mothers reported that optimal antenatal and postnatal care was provided by the health care workers as they gave prompt attention to patients, and rated these two services provided at 100% [17]. Put together, these two key factors (availability of skilled personnel and quality of care) could have operated to ensure that uptake of skilled delivery services in the Bongo district was high.

The WHO recognizes and supports a variety of birth environments, in the context of an organized health system and skilled attendance. The WHO in this regard does not recommend any particular setting. It however indicates that births can take place in appropriate places such as from the home to tertiary referral centres [18]. What remains critical is that the delivery is handled by skilled personnel, most of whom are found in health facilities.

Health services and facility use is to a large extent influenced by the characteristics of the health delivery system, accessibility, quality and cost of the services [19]. However, even where there is availability and a good supply of services, these services may not be fully used, and some women are more likely to use services than others [20]. The decision to seek skilled delivery services was found to be favourable in spite of the highly patriarchal nature of the region and district. The decision and subsequent choice of the delivery place (and in this study the choice to deliver at a health facility) was made by most of the women themselves (51%), with a few reporting that they did so in consultation with their partners (37%). This finding contradicts studies which indicate that many women in many communities in the African sub-region lack the decision making capacity to choose where to deliver and that this decision usually rests on the household head (mostly the husband), especially if such a decision would have some element of cost attached [14].

Several studies have emphasized factors such as cultural beliefs, socio-demographic characteristics, economic conditions, and physical and financial accessibility to be important determinants of the use of maternal health care services [6, 12, 13, 21]. This link between factors such as place of residence, maternal educational attainment, wealth status, ever use of contraception, complications with previous pregnancy and health insurance coverage have been well documented [2, 6, 14, 22]. This study found antenatal care to be significantly related to health facility delivery uptake. Frequency of ANC visits and satisfaction with ANC services provided all significantly related to health facility delivery. Facility delivery was high when ANC attendance was four times or more, a finding which is similar to one obtained in rural Cambodia [23]. Maternal mortality has also been found to be higher among women who attend ANC less than four times [24]. A study conducted by Anwar in Bangladesh [25] showed that frequent ANC influence choice of skilled care during delivery. Similarly, Zere found in Ghana that inaccessibility to ANC increases the odds of delivering without skilled personnel in attendance by 27 times and that increased ANC visits decreases the chance of delivering at home. It is documented that women who attend four or more ANC visits are more likely to deliver with professional assistance [26]. Attending ANC frequently on one hand has a positive effect on reducing maternal mortality as it helps in the early detection of obstetric conditions and on the other influences women’s decision to seek for skilled delivery assistance.

Having a valid national health insurance significantly associated with health facility delivery. NHIS membership was very high among the women and this could be attributed to the low economic status of women in the district. Ghana’s NHIS has a component that caters for health services including Maternal and Neonatal Care Health services which comes with, without out-of-pocket payments. Pregnant women with insurance, especially rural dwellers are therefore likely to opt for health facility delivery where skilled delivery service are provided [6]. This result is consistent with findings from other African countries where insurance-based programs and fee exemptions result in higher uptake of health facility delivery [6, 22]. A study in Kenya, for example, found that having insurance doubles the odds of a health facility delivery, which suggests that inability to pay for services is one of the major barriers to uptake of skilled delivery services [4].

### Limitations of the study

This study has some limitations and it is important to enumerate its key ones. First, the use of research assistants who were familiar to community members to collect data could have resulted in social desirability bias. Whiles we acknowledge this, we however anticipate that the use of community members created an atmosphere of trust with respondents which facilitated sincere responses. Second, since the interviews were conducted in the Frafra dialect, with the research assistants having to translate the responses back into the English language, we anticipate that some content may have been lost in translation. However, due to the close-ended nature of most of the questions this problem was minimized. Third, the questionnaire assessed the use of delivery health services a year preceding the survey which might have introduced recall bias. The aggregation of most of the participants in one group (skilled delivery) did not permit a binary logistic regression to further identify significant predictors or determinants of skilled delivery services utilization. We are also aware that the methodological nature of the cross-sectional study design limited the causality inference of the study variables. Lastly, generalizability of the study findings to the entire Ghanaian population is limited as the survey communities were all located within the Bongo district. Beyond generalization however, the findings of the study are encouraged.

## Conclusion

The UN Millennium Development Goal 5 which seeks to improve maternal health, stipulates two global targets. First, that maternal mortality ratio should be reduced by 75% between 1990 and 2015 and secondly, the universal coverage of skilled care at birth by 2015. Ghana as at the close of 2015 recorded an MMR of 319 per 100,000 live births [1], a figure which indicates insufficient progress made towards achieving the MDG 5 target of 185 per 100,000 live births. Although a Maternal Health Care Program which exempts women from paying for their care out of pocket upon confirmation of pregnancy following enrolment on NHIS now exists, the needed surge in the number of women in labour who utilize this free skilled assistance and care during labour is yet to materialize fully in the Bongo district (achieve 100% facility delivery) and Ghana at large. Despite its limitations, this study has served the purpose of highlighting critical areas at the household, community and district levels that could be streamlined in order to propel the quest for universal skilled delivery in the district forward.

To begin with, this study has revealed that antenatal care coverage in the Bongo district is high and appreciable. The 97.3% coverage observed for at least one antenatal visit before delivery is commendable. The 75% coverage observed for at least four visits can however be improved. More frequent contact with service providers during antenatal visits has the potential of influencing pregnant women to seek skilled assistance during labour and service providers must take full advantage of their interpersonal skills during the first initial contact to positively influence their clients to attend more frequently.

Health facility delivery was found to be very high in the district though the district is predominantly rural. This situation is very encouraging and draws emphasis on the fact the strategies currently in place to reduce maternal mortality and improve overall maternal health in the district is yielding great dividends. Educational attainment of women in the district was found to be very low, especially at the secondary and tertiary levels (upper echelons) and since mothers educational attainment was found to be significantly related to skilled delivery services utilization we recommend that advocacy on girl child education in the District be strengthened by the Ghana Education Service and women advocacy groups. Efforts should also be made by these stakeholders to see to enrolment, retention and transition of girls in school up to the secondary or tertiary level of education.

## Additional file

Additional file 1: Study Questionnaire: Factors influencing the utilization of skilled delivery services in Bongo District of Ghana. (DOCX 22 kb)

## Abbreviations

ANC: Antenatal care
CHPS: Community-based health planning and services
DHMT: District Health Management Team
GDHS: Ghana demographic health survey
GHS: Ghana health service
MDGs: Millennium development goals
MMR: Maternal mortality ratio
