# Supplementary material for: Factors associated with utilization of skilled service delivery among women in rural Northern Ghana: a cross sectional study
Source: BMC Pregnancy Childbirth. 2017 May 31;17:159. doi: 10.1186/s12884-017-1344-2 (PMC5452376; doi:10.1186/s12884-017-1344-2)
Supplement: Additional file 1: — Study Questionnaire: Factors influencing the utilization of skilled delivery services in Bongo District of Ghana. (DOCX 22 kb) [file 12884_2017_1344_MOESM1_ESM.docx]

**STUDY QUESTIONNAIRE**

**FACTOS INFLUENCING THE UTILIZATION OF SKILLED DELIVERY SERVICES IN BONGO DISTRICT OF GHANA**

Factors affecting the utilization of skilled delivery services in the Bongo District.

Interview with Mothers who delivered ≤ 1yr ago

Respondents Id No ………….. Location/Address…………………….

How old is your last child? …………………………………………….

Date of interview ………………………………………………………

**GENERAL CHARACTERISTICS**

1.1 Age …………………..

1.2 Parity (total No of births) …………………………

1.3 Marital status:

1: Single 2: Married 3: Widowed 4: Separated/divorced 5: Co-habiting

1.4 Educational Level

1: None 2: Primary 3: JSS 4: SSS 5: Tertiary

1.5 Occupation

1: Farmer 2: Trader 3: Professional 4: Skilled (specify) ………….

5: Unskilled (specify)……………… 6: Other (specify)……………………….

1.6 Ethnicity

1: Frafra2: Kusa 3: Gruni 4: Other (specify……………..

1.7 Religion

1: Christian 2: Moslem 3: Traditional 4: Others (Specify……………

1.8 What is the educational level of husband or spouse?

1: None 2: Primary 3: JSS 4: SSS 5: Tertiary

1.9 What is husband’s occupation?

1: Farmer 2: Trader 3: Professional 4: Skilled (specify) ………….

5: Unskilled (specify)……………… 6: Other (specify)……………

2.1 What is the main source of drinking water in this household?

1: Piped water inside 2: Piped water yard 3: Piped water public

4: Borehole/well 5: Stream/River 6: Water tanker 7: Rainwater tank

8: Sachet water 9: Bottled water

2.2 What type of toilet do you use in this house?

1: Flush toilet 2: Traditional pit toilet 3: Ventilated improved pit latrine

4: Bucket/pan 5: No facility/bush 6: Other (specify)………………………….

2.3 What is the main fuel used for cooking in this house?

1: Electricity 2: Gas 3: Kerosene 4: Charcoal 5: Wood

6: Other (specify)…………………..

2.4 Do you have any of the following? **(*multiple responses accepted*)**

1: A car 2: A refrigerator and/or freezer 3: Radio 4: Television(s)

5: Stove 6: telephone/cell phone

2.5 Are you currently a member of the National Health Insurance Scheme (NHIS?)

1: Yes 2: No

2.6 Source of income of main provider for the household?

1: Regular employment? 2: Irregular employment 3: Home employment

(income generating activity performed at home) 4: State pension

5: Contributions from others 6: Don’t know

7: Other (specify)………………………………….

2.7 How many people in all currently belong/live in this household? …………..

2.8 Can you kindly estimate the average monthly income in this household?

1: < ¢100 2: ¢100-¢299 3: ¢300-¢499 4: ¢500 or more

**ACCESS AND COST OF SEEKING MATERNITY AND ANTENATAL CARE**

3.1 How do you usually get to your nearest maternity home or clinic?

1: walk 2: taxi or bus 3: own car 4: other

3.2 How long does it normally take you to get to the nearest clinic or maternity home?

Minutes

3.3 How much does it cost to get to the nearest clinic? GHC

3.4 How do you usually get to the nearest Hospital?

1: walk 2: taxi or bus 3: own car 4: other (specify)……………

3.5 How long does it normally take you to get to the nearest hospital? (in minutes)

3.6 How much does it cost to get to the nearest hospital?

3.7 Did you receive or attend ANC during the last pregnancy/birth?

1: Yes 2: No (*If Yes proceed to 3.9 and If No to 4.6, then 5.1)*

3.8 If No can you briefly tell me why?

3.9 If Yes can you tell us briefly why?

4.0 If Yes where did you receive it from?

1: Hospital 2: Health center 3: Maternity home 4: TBA’s home

5: Other (specify

4.1 How many months pregnant were you when you first received or attended center for ANC?

1: <3months 2: b/n 3-6 months 3: >6months 4: Don’t know

4.2 How many ANC visits did you make before delivery?

1: One 2: Two 3: Three 4: Four or >

4.3 Were you seen by the one and the same person on all occasions of your ANC visit?

1: Yes 2: No 3: Don’t know

4.4 Were you satisfied with the reception or treatment you received at the place you attended your ANC?

1 Yes 2: No

4.5 Would you recommend ANC services at this place/person to other women?

1: Yes 2: No

Why or Why Not

**MATERNAL DELIVERY**

4.6 Where did you deliver your last baby?

1: Home 2: Hospital 3: Private clinic 4: Health Center

5: Maternity Home 6 TBA

**(*if Home delivery please jump to section 6.0)***

4.7 What type of delivery did you have at the health facility?

1: Vaginal 2: Vacuum 3: C/S 4 Other (specify)…………….

4.8 What was the outcome of the delivery at the health facility?

1: Live Baby 2: Still Birth

4.9 Did you have any problems getting to the health facility (when in labor) for the birth of your baby?

1: Yes 2: No

5.0 If Yes please briefly describe the nature of the problems (*probe and tick all that apply*).

1: Family consent or approval 2: Money needed for delivery 3: Getting a vehicle 4: Poor road network 5: Other (specify)…………….

5.1 Can you tell us who took the decision; you deliver at health facility and not at TBA/home? (probe)

1: My own preference/choice 2: Collective decision by me/spouse 3: Collective decision by family 4: Other (specify)…………..

5.2 At the health facility who attended to or assisted in your delivery process?

1: Doctor 2: Midwife 3: Nurse 4: Health care assistant 5: Other Staff (specify)………………

5.3 Were any of your relatives allowed to be with you during the delivery process?

1: Yes 2: No 3: Don’t know

5.4 Which would you prefer given the choice? To have relatives present to observe the delivery process.

1: Relatives present 2: Relatives absent 3: Don’t know

5.5 Did you have to pay any money for the last delivery?

1: Yes 2: No 3: Don’t know

5.6 If Yes how much money did you spend in all for the delivery? (direct [= formal charges] and indirect costs= informal)

1: Direct costs=GHC………. 2: Indirect costs =GHC……………………

5.7 If No who paid for the cost of the delivery?

1: NHIS 2: Other private insurance 3: Employer 4 Other (specify)………………….

5.8 How soon after the delivery were you discharged from the hospital?

1: Same day (<, 24hrs) 2: Day after (>24hrs)

5.9 Were you advised to come back to the health facility after the delivery?

1: Yes 2: No 3: Can’t remember

5.10 Now tell me how you were treated at by the health staff during the delivery? (probe and tick all that apply} 1: Good 2: Bad 3: Very bad 4: Normal

5.11 How would you rate the overall service rendered to you during the delivery process?

1: Poor 2: Average 3: Excellent

5.12 Would you recommend labor or delivery care at this health facility to other women?

1: Yes 2: No

Briefly explain to me why or why not………………………………………………

**6.0 FOR HOME DELIVERY ONLY**

6.1 What was the outcome of your delivery at home?

1: Live Baby 2: Still Birth

6.2 Are there any particular reasons why you decided to deliver at home? **(*Probe gently and tick all that apply)***

1: My own choice/preference 2: Collective family decision 3: Couldn’t make it to health facility (specify)……………. 4: Had transportation difficulties 5: Couldn’t afford facility charges 6: Didn’t like health facility 7: Traditional or cultural reasons (specify)

……… 8: Religious reasons (specify)………………… 9: Other (specify)……………..

6.3 Is /are there any persons who assisted you to deliver at home?

1: No I was alone 2: TBA 3: Health worker (nurse or HCW) 4: Midwife 5: Relative/friend/neighbour

6.4 Did you develop any problems or complications whist delivering at home?

1: Yes 2: No; If no go to 6.6

6.5 If Yes specify and briefly tell us what you did or were done for you.

……………………………………………………………………….

6.6 Tell me, is this the very first time that you are delivering at home?

1: Yes 2: No

**1: positive 2: negative**

6.7 If you got pregnant again and had to deliver, would you prefer a home or health facility?

1: Home 2: Health facility 3: Don’t know

6.8 Any particular reasons for this choice?

………………………………………………………………………………………………………………………………………………………………………………………………………………

**Thank you very much for the time spent with us!**
